# Presynaptic long-term plasticity

**DOI:** 10.3389/fnsyn.2013.00008

**Published:** 2013-10-17

**Authors:** Ying Yang, Nicole Calakos

**Affiliations:** ^1^Department of Pediatrics, Stanford University School of MedicineStanford, CA, USA; ^2^Departments of Neurology and Neurobiology, Center for Translational Neuroscience, Duke University Medical CenterDurham, NC, USA

**Keywords:** synaptic plasticity, neurotransmitter release, presynaptic plasticity, synaptic vesicle, long-term potentiation, long-term depression

## Abstract

Long-term synaptic plasticity is a major cellular substrate for learning, memory, and behavioral adaptation. Although early examples of long-term synaptic plasticity described a mechanism by which postsynaptic signal transduction was potentiated, it is now apparent that there is a vast array of mechanisms for long-term synaptic plasticity that involve modifications to either or both the presynaptic terminal and postsynaptic site. In this article, we discuss current and evolving approaches to identify presynaptic mechanisms as well as discuss their limitations. We next provide examples of the diverse circuits in which presynaptic forms of long-term synaptic plasticity have been described and discuss the potential contribution this form of plasticity might add to circuit function. Finally, we examine the present evidence for the molecular pathways and cellular events underlying presynaptic long-term synaptic plasticity.

## Introduction

Long-term synaptic plasticity is a fundamental property of the nervous system and is widely considered a primary mechanism for learning and memory (Kandel, 2001; Fusi et al., 2005). Long-term synaptic plasticity is defined by a long-lasting, activity-dependent change in synaptic efficacy. Long-term plasticity can bidirectionally modify synaptic strength—either enhancing (LTP, long-term potentiation) or depressing (LTD, long-term depression). Although early research focused on postsynaptic mechanisms, it is now clear that modification of synaptic strength may occur at either side of the synapse. Postsynaptic plasticity generally involves changes in postsynaptic receptor numbers or properties, while presynaptic plasticity involves an increase or decrease of neurotransmitter release.

Initial evidence for a presynaptic locus for long-term synaptic plasticity came from studies at hippocampal mossy fiber synapses and cerebellar parallel fiber synapses (Staubli et al., 1990; Zalutsky and Nicoll, 1990, 1992; Salin et al., 1996). In recent years, the list of brain regions found to express presynaptic LTP has markedly grown illustrating the prevalence of this form of synaptic plasticity. Moreover, the number of pathways implicated in the induction and expression has greatly expanded. Here we review the methodologies used to identify a presynaptic expression locus, describe the synapses where such plasticity exists, and discuss the molecular targets and putative cellular mechanisms responsible for presynaptic plasticity.

## Physiological characteristics supporting a presynaptic locus for long-term plasticity

In the last decade of the twentieth century, scientists debated passionately a seemingly simple question—whether the change in synaptic strength during long-term plasticity is due primarily to presynaptic alterations in neurotransmitter release or to postsynaptic modifications in receptor numbers and/or biophysical properties (Malinow and Tsien, 1990; Malinow, 1991; Stevens, 1993; Bear and Malenka, 1994; Isaac et al., 1996; Manabe, 1997). Strong arguments were made on both sides, with disagreements arising from reproducibility of experimental results (Kullmann and Siegelbaum, 1995; Nicoll and Malenka, 1999a) to interpretations of the same observations (Korn and Faber, 1998; Malenka and Nicoll, 1999). These disagreements in part reflect the fact that the same synapse may undergo various forms of plasticity, depending on induction protocols and receptor compositions at the time. They also illustrate the importance of recognizing assumptions commonly held with particular methods used to obtain the experimental observations. In many cases, electrophysiological measurement of postsynaptic responses was the readout from which it was necessary to infer the nature of the synaptic change. This inference included assumptions of synaptic properties that broke down as exceptions were realized. In this section, we will review the general methodologies that have been employed to identify a presynaptic locus. We will discuss the basic assumptions and limitations of these methods and introduce more recent technical advances for monitoring neurotransmitter release and its alteration during long-term plasticity.

### Evidence using postsynaptic activity as the reporter

Initial attempts to dissect pre- or post-synaptic functions during long-term plasticity utilized conventional electrophysiological techniques. The approaches used generally relied upon the postsynaptic response as a reporter of synaptic activity, and typically included the aggregate response of a few to a few hundred synapses.

#### Quantal analysis, coefficient of variation and minimal stimulation

The idea of using quantal analysis to identify presynaptic mechanisms was proposed by Sir Bernard Katz (1971), following the discoveries of spontaneously occurring miniature synaptic potentials, or quanta, and the observation that evoked postsynaptic responses correspond to integral multiples of the quantal unit (Fatt and Katz, 1952; Kuno, 1964). Since its introduction, quantal analysis and its derivatives, such as coefficient of variation (*CV*) and minimal stimulation, have been used widely to investigate whether a change in neurotransmitter release accompanies long-term changes in synaptic strength. These methods were used in the first studies that proposed a presynaptic expression locus of LTP (at hippocampal mossy fiber-CA3 synapses) (Malinow and Tsien, 1990; Hirata et al., 1991), and have been subsequently employed to support a presynaptic expression locus during long-term plasticity in diverse brain regions which include hippocampal mossy fiber-interneuron synapses (Pelkey et al., 2005), hippocampal GABAergic synapses (Laezza et al., 1999), the amygdala (Tsvetkov et al., 2002; Fourcaudot et al., 2008), the neocortex (Torii et al., 1997; Sjöström et al., 2003, 2004; Huang et al., 2008a; Sarihi et al., 2012), striatum (Choi and Lovinger, 1997a), ventral tegmental area (VTA) (Pan et al., 2008) and cerebellum (Maejima et al., 2001).

The quantal analysis hypothesis proposes that the average amplitudes and fluctuations of postsynaptic responses follow simple or compound binomial statistics, which is supported by experimental evidence (Redman, 1990; Korn and Faber, 1991; Stevens, 1993). Under the simple binomial model, the average release probability, *Pr*, is assumed to be the same at all synapses. The *CV* is determined by the number of release sites, *n*, which is approximated by the number of synapses for most synapses in the CNS (Korn and Faber, 1991), and the average release probability, Pr, but is independent of quantal size, *q*, or the size of the postsynaptic response to any quanta of neurotransmitters^1^. Specifically,

$$CV^{2}=(1-Pr)/nPr$$

Assuming a constant number of synapses, *CV*^2^ is negatively correlated with *Pr*—when *Pr* increases, *CV*^2^ will decrease and vice versa. In this way, a change in *CV* can be monitored as an indicator for changes in presynaptic release probability.

In minimal stimulation experiments, stimulation intensity is adjusted to a minimal level such that one or only a few presynaptic fibers are activated, and presumably only one or a few presynaptic release sites receive action potentials. Due to the stochastic nature of vesicle release (Katz, 1971), postsynaptic responses are not elicited with every stimulation. Failure rate, the average rate of not observing a response over multiple trials, is determined by the number of activated synapses, n, and the average release probability of these synapses, Pr,

$$R_{\text{Failure}}={\left(1-Pr\right)}^{n}$$

Assuming a constant number of synapses, the failure rate is negatively correlated with average release probability, *Pr*. Therefore, failure rate in minimal stimulation experiments serve as an index of average release probability, with a higher failure rate corresponding to a lower release probability (Malinow and Tsien, 1990; Bekkers and Stevens, 1991).

As stated above, one of the assumptions for using a change in *CV* or failure rate to indicate a change in release probability is that the number of release sites remains constant. However, this assumption was called into question by the discovery of “silent synapses” (Malinow, 1991; Isaac et al., 1995; Liao et al., 1995). These synapses lack functional AMPA receptors but contain functional NMDA receptors postsynaptically. They were referred to as “silent” because at typical resting membrane potentials, NMDA receptors are blocked by magnesium and would not participate in synaptic responses under typical recording conditions. Changing the number of silent synapses is thus a postsynaptic mode of functionally changing, n, the number of release sites. In *CV* and minimal stimulation experiments, these synapses would not contribute to the initial number of release sites, *n*. However, upon LTP induction, functional AMPA receptors are recruited and inserted into the membrane of silent synapses and now contribute to the postsynaptic response. This postsynaptic phenomenon results in a decrease in *CV*^2^ and a decrease in failure rate. Without consideration of this possibility, the interpretation of these data would be (and was) that an increase in Pr had occurred, supporting the idea of presynaptic mechanisms for long-term plasticity (Malinow and Tsien, 1990; Bekkers and Stevens, 1991). In the early nineties, the expression locus for NMDA receptor-dependent LTP at hippocampal Schaffer collateral-CA1 synapses was debated fiercely. The accompanying decrease in *CV*^2^ and failure rate was viewed as support for a presynaptic locus (Malinow and Tsien, 1990; Bekkers and Stevens, 1991; Bolshakov and Siegelbaum, 1995). However, doubts started to emerge when it was noted that the relative contributions of AMPA and NMDA receptors to the postsynaptic response were also changing (Malinow and Tsien, 1990; Isaac et al., 1995; Kullmann and Siegelbaum, 1995). The subsequent discovery of postsynaptically silent synapses provided the necessary mechanistic insight to resolve these apparent discrepancies (Isaac et al., 1995; Liao et al., 1995). Postsynaptic unsilencing after LTP clearly reveals the postsynaptic expression locus of NMDA receptor-dependent LTP at hippocampal Schaffer collateral-CA1 synapses, with the exception of certain induction protocols (Zakharenko et al., 2002; Bayazitov et al., 2007) or in developing hippocampus (Bolshakov and Siegelbaum, 1995; Palmer et al., 2004).

To summarize, while quantal analysis and its derivatives (*CV*^2^ and failure rate) provide evidence for presynaptic long-term plasticity, they cannot stand alone as the sole evidence. The examples discussed above emphasize the importance of understanding the inherent assumptions underlying each approach to accurately guide interpretation and selection of complementary experiments.

#### Miniature postsynaptic currents and strontium evoked asynchronous postsynaptic currents (mini analysis)

A second experimental approach based on quantal analysis is examination of quantal synaptic events, namely, miniature postsynaptic currents and strontium evoked asynchronous postsynaptic currents. Miniature postsynaptic currents are useful when the majority of synapses assayed undergo plasticity, and asynchronous postsynaptic currents allows for analysis from the subset of synapses being stimulated (Oliet et al., 1996; Choi and Lovinger, 1997b). These methods have been employed to support a presynaptic expression locus in metabotropic glutamate receptor-dependent LTD at hippocampal Schaffer collateral-CA1 synapses (Oliet et al., 1997), and in the striatum (Choi and Lovinger, 1997b), as well as endocannabinoid-dependent LTD in the amygdala (Robbe et al., 2002b; Azad et al., 2004) and prefrontal cortex (Lafourcade et al., 2007). Early studies found that changing external calcium concentration, a manipulation known to alter presynaptic neurotransmitter release probability, changes the frequency of quantal events (del Castillo and Katz, 1954; Dodge and Rahamimoff, 1967). Therefore, a primary change in the frequency of quantal events is considered to represent a change in presynaptic neurotransmitter release probability (Choi and Lovinger, 1997b). However, this interpretation is also challenged by the existence of silent synapses. As reasoned above, postsynaptic unsilencing/silencing may increase/reduce the number of functional synapses as measured by postsynaptic currents, altering the frequency of quantal responses (Nicoll, 2003). Therefore, quantal analysis, too, cannot be used as the sole evidence for a presynaptic alteration. Though, consideration of the priniciples and assumptions underlying each assay can help in the selection of complementary assays with non-overlapping caveats.

#### Release probability monitored by progressive irreversible blockade of NMDA receptor-mediated synaptic responses (MK801 blockade)

MK801 is an irreversible, open channel blocker of the NMDA receptor (NMDAR) (Hessler et al., 1993; Rosenmund et al., 1993). Repeated activation of synapses in the presence of MK801 results in progressive decline of the NMDAR–mediated current, as each stimulus releases glutamate and opens NMDAR channels, the receptor becomes irreversibly blocked upon MK801 binding and thus are unavailable for subsequent responses. The rate of decline of the evoked NMDAR-mediated EPSC depends on the average release probability, Pr, because a higher Pr results in more frequent presynaptic release and consequently more rapid blockade of NMDARs. The rate of MK801 blockade of NMDAR-mediated current has been used to show that an increase in release probability is associated with LTP at mossy fiber-CA3 pyramidal synapses (Weisskopf and Nicoll, 1995), and that a decrease in release probability occurs with metabotropic glutamate receptor (mGluR)-mediated LTD at CA3-CA1 synapses of neonatal rats (Nosyreva and Huber, 2005). While this approach is not confounded by silent synapses, other limitations exist. First, this method relies upon the assumption that the activity of postsynaptic NMDARs is not altered as a result of the induction protocol. Should NMDAR plasticity exist and be unrecognized, the interpretation would erroneously attribute the changes to presynaptic release probability. Second, because MK801 blockade is irreversible, the same pool of synapses cannot be tested repeatedly, for example, before and after induction of LTP. Due to this limitation, evidence for a change in release probability is surmised by comparing the rate of blockade at a second set of stimulated synapses that have not undergone plasticity induction. In this condition, the assumption that the second set of stimulated afferents activates synapses with similar release probabilities must hold, and may not always be the case (Rosenmund et al., 1993; Dobrunz and Stevens, 1997). Third, if plasticity involved the addition of newly active presynaptic release sites but with the same release probability, the rate of MK801 blockade would not detect such a change (Malinow, 1994). Finally, this approach is only suitable for glutamatergic synapses.

#### Paired-pulse ratio (PPR)

When two stimuli are delivered to a presynaptic axon in rapid succession (fractions of a second), the second postsynaptic response often differs characteristically from the first. For example, being repeatedly larger or smaller by a certain degree. The ratio of the amplitude of the second EPSC response to that of the first is called the paired-pulse ratio (PPR). In the mammalian central nervous system, PPR inversely correlates with release probability (Zucker, 1989; Debanne et al., 1996; Dobrunz and Stevens, 1997; Zucker and Regehr, 2002) and is regularly used as an indicator of release probability. The mechanism for this change has been attributed to effects of presynaptic calcium dynamics and depletion of “release-ready” vesicles (Zucker, 1989; Debanne et al., 1996; Inchauspe et al., 2004; Catterall and Few, 2008). A decrease in PPR accompanies LTP at mossy fiber-CA3 pyramidal synapses (Zalutsky and Nicoll, 1990), corticothalamic synapses (Castro-Alamancos and Calcagnotto, 1999), cortical and thalamic synapses with lateral amygdala (Tsvetkov et al., 2002; Samson and Paré, 2005) and in other brain regions (Li et al., 2000; Lauri et al., 2007; Sarihi et al., 2012). Conversely, an increase in PPR accompanies LTD from electric stimulation or drug applications in various brain regions (Huang et al., 2001; Gerdeman et al., 2002; Chevaleyre and Castillo, 2003; Nosyreva and Huber, 2005). One caveat to the use of PPR as support for a presynaptic mechanism of plasticity is that changes in PPR are not exclusively mediated by changes in presynaptic release probability. For example, PPR can be influenced by postsynaptic receptor desensitization and lateral diffusion (Trussell et al., 1993; Heine et al., 2008; Frischknecht et al., 2009; Opazo et al., 2010). Additionally, modifications of short-term plasticity may occur that are not associated with a change in release probability but do affect PPR (Geppert et al., 1997; Basu et al., 2007; Shin et al., 2010b). These limitations again place PPR in the toolbox of reagents to suggest a presynaptic basis, but render this approach insufficient on its own to prove such a mechanism.

#### AMPA: NMDA ratio

AMPA receptors and NMDA receptors are separately regulated at postsynaptic terminals. Forms of postsynaptic long-term plasticity, for example LTP involving delivery of AMPAR but not NMDAR to postsynaptic sites, commonly alter the ratio of activity between these two receptor types. Conversely, proportional changes in AMPA receptor-mediated and NMDA receptor-mediated responses have been used to support a presynaptic basis for long-term plasticity. Observations of a change in synaptic strength with a stable AMPA:NMDA ratio have been reported at hippocampal CA3-CA1 synapses and mossy fiber-interneuron synapses as supplemental evidence for a presynaptic locus of expression after long-term plasticity (Bayazitov et al., 2002; Lei and McBain, 2004). Conversely, if the population of presynaptic terminals being monitored does not have uniform AMPA:NMDA ratios, enhancing a fraction of synapses with distinct properties could change this ratio even though the mechanism was presynaptic.

#### Excitatory postsynaptic calcium transients (EPSCaTs)

The aforementioned methods are all based on electrophysiological analysis, which reports simultaneous activity of an unknown and potentially large number of synapses. As stated before, synapses from the same postsynaptic cell do not always share the same release probability presynaptically (Rosenmund et al., 1993; Dobrunz and Stevens, 1997). This heterogeneity in release probability may create artifacts in some scenarios while reduce the ability to detect a presynaptic alteration in others. To minimize interpretational difficulties, optical methods have been employed to provide single synapse resolution.

At an individual dendritic spine of an excitatory synapse, a calcium transient may be detected in response to a single afferent stimulus (Emptage et al., 1999; Oertner et al., 2002; Emptage et al., 2003; Reid et al., 2004; Enoki et al., 2009). Such events have been termed “EPSCaTs” for excitatory postsynaptic calcium transients. The probability of observing an EPSCaT at a synapse correlates with release probability at hippocampal CA3-CA1 and mossy fiber synapses (Emptage et al., 1999; Oertner et al., 2002; Emptage et al., 2003; Reid et al., 2004; Enoki et al., 2009), and the amplitude of EPSCaTs positively correlates with the amplitudes of excitatory postsynaptic potential (EPSP) at mossy fiber-CA3 pyramidal synapses (Reid et al., 2004). Monitoring EPSCaTs in rat organotypic hippocampal cultures, Emptage et al. (2003) showed that LTP at CA3-CA1 synapses is associated with a decrease in failure rate at single synapses, supporting the involvement of a presynaptic locus of expression. This approach overcomes limitations imposed by the presence of synapse unsilencing because the same active synapses are monitored before and after plasticity induction. Using this method, Reid et al. (2004) observed an increase in EPSCaT size in addition to a decrease in failure rate upon LTP induction at mossy fiber-CA3 pyramidal synapses, suggesting that LTP expression at single synapse may involve multi-vesicular release in addition to an increase in release probability.

EPSCaTs significantly advance the study of synaptic plasticity expression mechanisms by allowing interrogation of an individual synapse over time. Yet this approach has some limitations. For those spines showing an EPSCaT signal only after LTP induction (Reid et al., 2004), EPSCaT cannot distinguish whether the unsilencing reflects insertion of receptors postsynaptically or activation of new functional release sites presynaptically. Further, since the calcium transients include calcium influx through voltage-gated calcium channels (Yuste et al., 1999; Reid et al., 2001), the size of an EPSCaT could be altered by a modulation of postsynaptic calcium channel activity and/or back propagating action potentials (Yuste et al., 1999). If such mechanisms occurred, interpretations assigning amplitude changes to changes in the quanta of neurotransmitter released presynaptically would be erroneous. While the EPSCaT approach constitutes a major advance by allowing visualization of activity at single synapses, the main drawback of this approach is that the signal again arises from postsynaptic activity making it difficult to exclude with certainty contributions due to postsynaptic alterations.

### Evidence measured from the presynaptic terminal

Given the limitations of inferring presynaptic changes through a readout involving the postsynaptic apparatus, newer methodologies have been developed to allow direct measurement of presynaptic neurotransmitter release. Early approaches used fluorescent lipophilic dyes (FM dyes) to image presynaptic vesicles (Stanton et al., 2001; Zakharenko et al., 2001). PHluorins, pH-sensitive GFP molecules fused to synaptic vesicle proteins (Miesenböck et al., 1998), were later employed to address the same question (Bayazitov et al., 2007).

#### FM dyes

FM dyes were first developed by Fei Mao (hence, FM) to image synaptic vesicle recycling (Betz and Bewick, 1992). These dyes are not fluorescent in aqueous solutions but become fluorescent when associated with lipids. Synaptic vesicles are loaded with dye by a protocol that stimulates high volumes of vesicle fusion and recycling typically using 10–12 K stimuli at 10 Hz (Harata et al., 2001; Zakharenko et al., 2001; Blundon and Zakharenko, 2008; Park et al., 2012). In this way, the inner leaflet of the synaptic vesicle lipid bilayer becomes loaded with dye. Because the dye cannot traverse the lipid bilayer, once a synaptic vesicle is loaded, it can only lose its fluorescence by exocytosis which allows the dye to diffuse away. This technique has been used to measure release probability at single synapses by monitoring the destaining rate of FM dyes in response to repeated axonal stimulation (Zakharenko et al., 2001). Using this methodology to determine whether presynaptic release probability was altered as a result of long-term plasticity, differences in FM dye destaining rates were observed before and after both LTP and LTD induction at CA3-CA1 synapses in hippocampal slices (Stanton et al., 2001; Zakharenko et al., 2001, 2002, 2003).

While the application of the FM assay is a great step forward for directly monitoring the presynaptic component, several practical limitations prevent its widespread utility for studying mechanisms of long-term synaptic plasticity. First, the populations of synaptic vesicles that are loaded must be reproducible across conditions being compared. There are multiple intracellular pools of synaptic vesicles (e.g., reserve pool, readily releasable pool). Changes in the distribution of newly endocytosed (and thus dye-loaded) vesicles among these pools could alter observed destaining rates independent of an effect on release probability (Maeno-Hikichi et al., 2011). Second, FM dyes require a few seconds to diffuse away from synaptic vesicles, as such, their destaining rate fails to reflect vesicle exocytosis under conditions of rapid vesicle recycling or kiss and run events (Maeno-Hikichi et al., 2011). Third, FM dyes cannot be used for continuous monitoring of presynaptic release because the dye is lost to the extracellular medium upon exocytosis. Thus, for experiments studying long-term plasticity, the loading procedure has to be repeated. This necessity raises another potential caveat because the loading protocol [typically 10 Hz trains for several minutes (Harata et al., 2001; Zakharenko et al., 2001; Blundon and Zakharenko, 2008; Park et al., 2012)] may induce its own plasticity, possibly occluding further plasticity. Mossy fiber-CA3 synapses are one example of synapses that are modified by 10 Hz activity (Nicoll and Schmitz, 2005).

#### pHluorins

The pHluorins are pH-sensitive GFP molecules that lose fluorescence as pH decreases. Exploiting the fact that synaptic vesicles maintain their lumen at a low pH, Miesenböck et al. (1998) linked pHluorins to a vesicle membrane protein to report transmission at individual presynaptic terminals. SynaptopHluorin (spH), a fusion protein of VAMP2/synaptobrevin and pHluorin, exhibits little fluorescence inside acidic synaptic vesicles, but increases its fluorescence when the synaptic vesicle lumen is exposed to the extracellular space during exocytosis. One advantage of this approach is that the dye can be used to monitor vesicle over multiple rounds of exo-/endo-cytosis because it is an integral membrane component of the vesicle. The pHluorin-based indicators are genetically encoded, and transgenic mice expressing these indicators have been developed (Araki et al., 2005; Li et al., 2005; Tabares et al., 2007). Using a transgenic mouse that expresses spH in CA3 pyramidal cells, Bayazitov et al. (2007) monitored presynaptic vesicle exocytosis for several hours while simultaneously measuring postsynaptic potentials. Because fluorescence from single vesicle fusion events could not be reliably monitored, presynaptic release probability was inferred from changes in the peak fluorescence in response to a test stimulus train (10 Hz for 5 s). Using this approach, Bayazitov et al. concluded that CA3-CA1 LTP induced by 200 Hz tetanization or theta-burst stimulation consisted of slow presynaptic and fast postsynaptic components. Enhancement of postsynaptic potentials (without changes in peak evoked spH fluorescence) was rapid (around 1 min); whereas changes in peak spH fluorescence in response to the test train increased gradually and stabilized approximately 90 min after induction. These experiments provide evidence that both presynaptic and postsynaptic modifications can be involved in the expression of LTP, albeit at different time scales.

An obvious limitation of this approach is the inability to monitor a single release event. Instead, conclusions rely on an inference of the average release probability derived from the behavior of a population of vesicles stimulated during a moderate frequency “test” train. Again, because the test train itself may induce short-term plasticity, it is difficult to distinguish whether observed differences in fluorescence are due to long-term changes in release probability or short-term plasticity effects. Newer generation pHluorins appear to be overcoming this obstacle. The pHluorins have been fused to two other synaptic vesicle proteins, vGluT1 and synaptophysin (Granseth et al., 2006; Voglmaier et al., 2006). These proteins both demonstrate significantly lower surface expression and enhanced signal-to-noise ratio relative to spH (Balaji and Ryan, 2007; Zhu et al., 2009). Both proteins have been proven useful for detecting single release events in cultured neurons (Balaji and Ryan, 2007; Matz et al., 2010). In principle these tools could be used in brain slice preparations to study mechanisms of long-term synaptic plasticity. More recent advances, such as a molecular glutamate sensor which has been used in tissue preparations and has excellent signal-to-noise properties (Marvin et al., 2013) or biotinylated vesicular neurotransmitter reporters (Xu et al., 2013), also show great promise for directly monitoring presynaptic function. Finally, optogenetic techniques (Boyden et al., 2005) can also be employed to achieve activation of single presynaptic boutons to facilitate the study of presynaptic plasticity mechanisms.

In summary, there is a wide array of conventional electrophysiological and newer imaging technologies that is valuable for examining presynaptic mechanisms of synaptic plasticity. Because no assay is sufficient to stand on its own to definitively implicate the presynaptic site, these are best used in combinations with the least overlapping caveats to support presynaptic plasticity mechanisms. In Table 1, we provide a summary of the assays that have been used to support a presynaptic locus for long-term synaptic plasticity in various brain regions.

**Table 1:** **Presynaptic long-term plasticity is a widespread phenomenon in the brain**.

**Brain region**	**Synapses**	E/I^*^	**LTP/LTD**	**Methods**
Hippocampus	Mossy fiber-CA3 synapses	E	LTP	CV analysis (Hirata et al., 1991; Xiang et al., 1994)
				Failure rate (Xiang et al., 1994; Maccaferri et al., 1998)
				PPR (Zalutsky and Nicoll, 1990; Xiang et al., 1994)
				MK801 blockade (Weisskopf and Nicoll, 1995)
				EPSCaT (Reid et al., 2004)
			LTD/de-potentiation	CV analysis (Domenici et al., 1998; Huang et al., 2002)
				Failure rate (Domenici et al., 1998; Maccaferri et al., 1998; Huang et al., 2002)
				MK801 blockade (Huang et al., 2002)
	Mossy fiber-interneuron synapses	E	LTD	CV analysis (Lei and McBain, 2004; Pelkey et al., 2005)
				Failure rate (Pelkey et al., 2005)
				PPR (Lei and McBain, 2004; Pelkey et al., 2005)
			De-depression^**^	CV analysis (Pelkey et al., 2005)
				Failure rate (Pelkey et al., 2005)
				PPR (Pelkey et al., 2005)
	CA3-CA1 Schaffer collateral synapse	E	LTP	CV analysis (Sokolov et al., 2002)
				Failure rate (Malinow, 1991)
				EPSCaT (Emptage et al., 2003)
				FM dye (Zakharenko et al., 2002)
				spH (Bayazitov et al., 2007)
			LTD	CV analysis (Fitzjohn et al., 2001)
				Failure rate (Fitzjohn et al., 2001)
				PPR (Fitzjohn et al., 2001; Faas et al., 2002; Zhang et al., 2006)
				FM dye (Zakharenko et al., 2002; Stanton et al., 2003)
			eCB-LTD (immature hippocampus)	CV analysis (Yasuda et al., 2008)
	Interneuron-CA1 synapse	I	eCB-LTDi, heterosynaptic (Schaffer collateral input)	Failure rate (Chevaleyre et al., 2007)
				Mini analysis (Chevaleyre et al., 2007)
				PPR (Chevaleyre and Castillo, 2003; Chevaleyre et al., 2007)
Cerebellum	Parallel fiber-Purkinje cell synapses	E	LTP	CV analysis (Bender et al., 2009)
				PPR (Salin et al., 1996; Bender et al., 2009)
	Parallel fiber-stellate cell synapses	E	eCB-LTD	Failure rate (Soler-Llavina and Sabatini, 2006)
				PPR (Soler-Llavina and Sabatini, 2006)
	Stellate cell-stellate cell synapses	I	LTP, heterosynaptic (parallel fiber input)	CV analysis (Lachamp et al., 2009)
				Failure rate (Lachamp et al., 2009)
				Mini analysis (Lachamp et al., 2009)
				PPR (Lachamp et al., 2009)
Thalamus	Cortical-thalamic synapses	E	LTP	PPR (Castro-Alamancos and Calcagnotto, 1999)
Hypothalamus	Inhibitory synapses in dorsomedial hypothalamus	I	eCB-LTDi, heterosynaptic	CV analysis (Crosby et al., 2011)
				PPR (Crosby et al., 2011)
			NO-LTPi, heterosynaptic	CV analysis (Crosby et al., 2011)
				PPR (Crosby et al., 2011)
Amygdala	Cortical-lateral amygdala synapse	E	LTP, homosynaptic and heterosynaptic (thalamic input)	CV analysis (Tsvetkov et al., 2002; Humeau et al., 2003)
				Failure rate (Tsvetkov et al., 2002)
				PPR (Huang and Kandel, 1998; Tsvetkov et al., 2002; Humeau et al., 2003)
				MK801 blockade (Shaban et al., 2006)
	Thalamic-lateral amygdala synapse	E	LTP	Failure rate (Shin et al., 2010b)
				PPR (Shin et al., 2010b)
	Inhibitory synapses in basolateral nucleus	I	eCB-LTDi, heterosynaptic	Mini analysis (Azad et al., 2004)
				PPR (Marsicano et al., 2002)
Striatum	Excitatory synapses onto MSNs	E	eCB-LTD	PPR (Gerdeman et al., 2002; Kreitzer and Malenka, 2005)
	Inhibitory synapses onto MSNs	I	eCB-LTDi, heterosynaptic	Mini analysis (Adermark et al., 2009)
Nucleus accumbens	Excitatory synapses onto MSNs	E	LTD (pre mGluR2/3 LTD and eCB-LTD)	Mini analysis (Robbe et al., 2002a,b)
				PPR (Robbe et al., 2002a)
Cortex	Visual cortex, L5-L5	E	eCB-LTD	CV analysis (Sjöström et al., 2003, 2004)
				PPR (Sjöström et al., 2004),
	Visual cortex, FSI-FSI synapses, L2/3-L2/3	I	LTP	CV analysis (Sarihi et al., 2012)
				PPR (Sarihi et al., 2012)
	Somatosensory cortex, L4-L2/3	E	eCB-LTD	PPR (Bender et al., 2006)
	Prefrontal cortex, L2/3-L6	E	eCB-LTD	CV analysis (Lafourcade et al., 2007)
				Mini analysis (Lafourcade et al., 2007)
Ventral tegmental area	Excitatory synapses onto dopamine neurons	E	eCB-LTD	CV analysis (Haj-Dahmane and Shen, 2010)
				Failure rate (Haj-Dahmane and Shen, 2010)
	Inhibitory synapses onto dopamine neurons	I	eCB-LTDi, heterosynaptic	Mini analysis (Pan et al., 2008)
			NO-LTPi, heterosynaptic	PPR (Nugent et al., 2007)
Dorsal cochlear nucleus	Excitatory synapses onto Cartwell cells	E	eCB-LTD	CV analysis (Tzounopoulos et al., 2007)
				PPR (Tzounopoulos et al., 2007)
Superior colliculus	Cortical-tectal inhibitory synapses	I	eCB-LTDi, heterosynaptic	PPR (Henneberger et al., 2007)

* E, excitatory; I, inhibitory.

** De-depression at mossy fiber-interneuron synapses can only occur after expression of LTD and internalization of surface mGluR7b receptors. In naïve slices, LTP cannot be induced at these synapse.

MSN, medium spiny neuron; FSI, fast-spiking interneuron.

## Forms of presynaptic long-term plasticity

**Figure 1:**
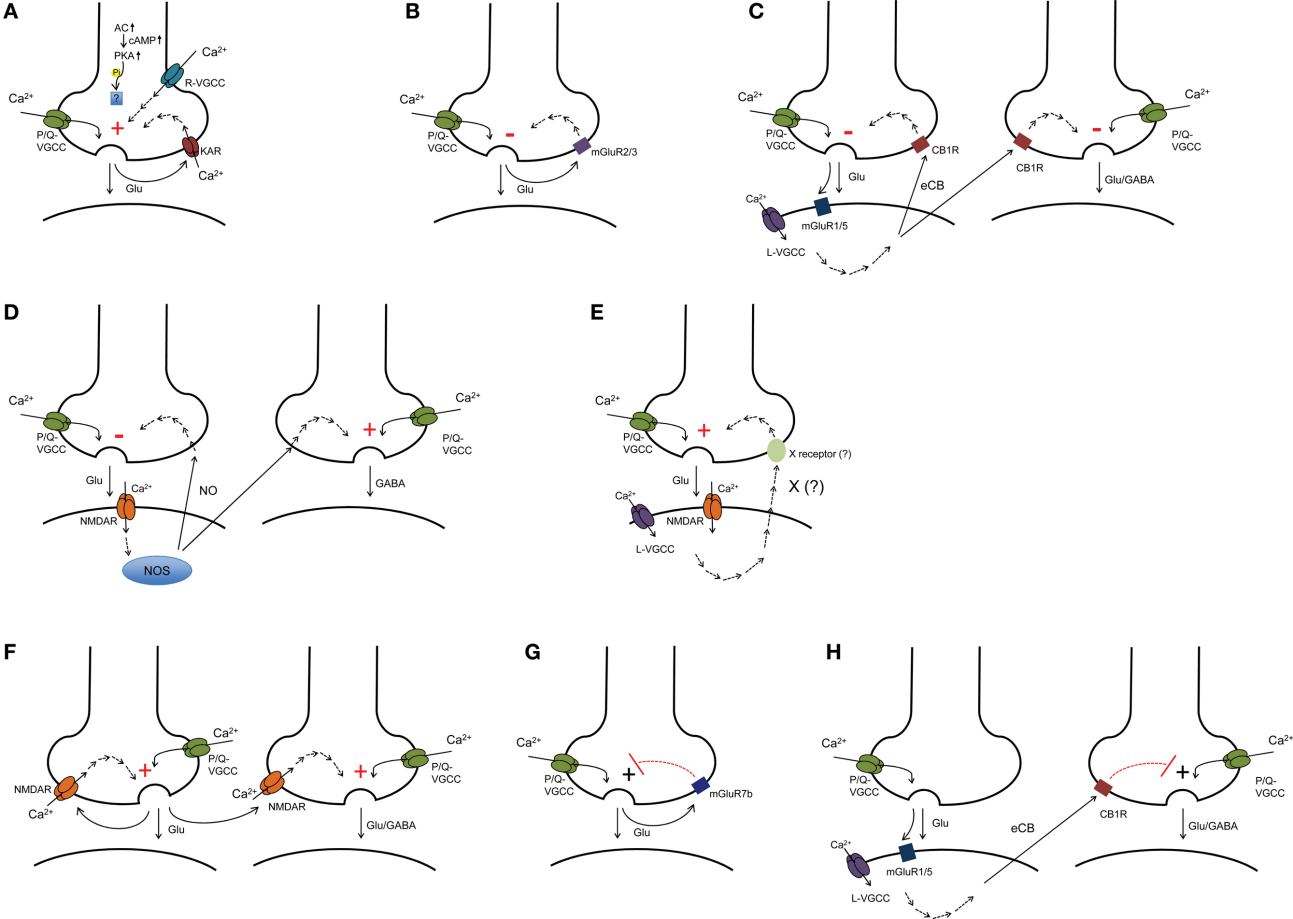
**Induction mechanisms identified for presynaptic LTP and LTD. (A,B)** presynaptic plasticity induced presynaptically (e.g., mossy fiber LTP and LTD). **(C)** eCB-dependent homosynaptic and heterosynaptic LTD. **(D)** NO-dependent LTD at excitatory synapses and LTP at inhibitory synapses. **(E)** postsynaptically induced LTP with as yet unidentified retrograde signaling mechanism (e.g., CA3-CA1 LTP). **(F)** presynaptic NMDAR-dependent homosynaptic and heterosynaptic LTP (e.g., LTP at cortico-LA synapses). **(G)** presynaptic LTP gated by mGluR7b at mossy fiber-SLIN synapses. **(H)** presynaptic LTP gated by CB1R (e.g., LTP at thalamic-LA synapses). AC, adenylate cyclase; cAMP, cyclic adenosine monophosphate; PKA, protein kinase A; VGCC, voltage-gated calcium channel; KAR, kainate receptor; mGluR, metabotropic glutamate receptor; eCB, endocannabinoid; CB1R, cannabinoid receptor type 1; NO, nitric oxide; NOS, nitric oxide synthase; CP-AMPAR SLIN, calcium permeable-AMPA receptor containing stratum lucidum interneurons.

In this section, we will introduce the particular synapses at which presynaptic forms of long-term plasticity have been documented. We will review the evidence that supports a presynaptic expression locus and discuss the potential functional significance of presynaptic long-term plasticity at these synapses. We will also review briefly the induction mechanism (Figure 1); however, for a detailed discussion of induction requirements, please see two outstanding recent reviews by Castillo et al. (2011) and Castillo (2012).

### Presynaptically induced and expressed long-term plasticity

Hebbian forms of synaptic plasticity are well described and have an intuitive appeal as to their role in learning. However, not all forms of long-term synaptic plasticity have properties consistent with Hebbian learning (which postulates a coordinated response of the postsynaptic site based upon the activity of a presynaptic afferent). For example, when long-term synaptic plasticity is both induced and expressed at the presynaptic terminal, Hebbian rules are violated. In these forms of plasticity, activation of the presynaptic neuron will alter the synaptic weight at all of its postsynaptic contacts, independent of postsynaptic coordinated activity. In this type of plasticity, the signaling efficacy of one presynaptic neuron may be increased relative to other presynaptic neurons. These forms of plasticity may be important for efficient circuit re-organization.

#### LTP and LTD at hippocampal mossy fiber-CA3 synapses

Mossy fiber synapses, the synapses between the axons of dentate granule cells and the proximal apical dendrites of CA3 pyramidal neurons, were the first synapses at which presynaptic long-term plasticity was described (Zalutsky and Nicoll, 1990; Kobayashi et al., 1996). Both LTP and LTD of mossy fiber-CA3 synapses are expressed by a change in release probability (*Pr*), as evidenced by quantal analysis, MK801 blockade of NMDA receptor-mediated current and PPR data (Zalutsky and Nicoll, 1990; Hirata et al., 1991; Xiang et al., 1994; Weisskopf and Nicoll, 1995; Domenici et al., 1998; Maccaferri et al., 1998). In addition to an increase in synaptic release probability, data from EPSCaTs imaged in slice cultures show that presynaptic unsilencing and an increase in quantal content may also occur (Reid et al., 2004). A presynaptic de-potentiation following LTP has also been described at these synapses and appears to follow a process similar to that involved in LTD (Tzounopoulos et al., 1998; Huang et al., 2002).

Induction of presynaptic long-term plasticity at these synapses requires action potential firing and calcium influx at the granule cell soma (Barnes et al., 2010), and calcium entry through calcium channels at the presynaptic terminal (Castillo et al., 1994; Domenici et al., 1998; Tzounopoulos et al., 1998; Kobayashi et al., 1999). Although no specific type of calcium channel is indispensable for plasticity induction (Castillo et al., 1994; Breustedt et al., 2003), the threshold for LTP induction is lowered by calcium influx through R-type calcium channels (Breustedt et al., 2003; Dietrich et al., 2003) or presynaptic kainate receptors and subsequent calcium release from intracellular calcium stores (Bortolotto et al., 1999; Lauri et al., 2003; Pinheiro et al., 2007). LTP induction has also been shown to require vesicular zinc and TrkB activation (Lu et al., 2000; Li et al., 2001; Huang et al., 2008b; Pan et al., 2011; but see Vogt et al., 2000; Matias et al., 2006). LTD induction requires activation of type II metabotropic glutamate receptors, mGluR 2/3, at presynaptic terminals (Yokoi et al., 1996; Tzounopoulos et al., 1998; Huang et al., 1999; Nicholls et al., 2006). Finally, two features that are commonly involved in postsynaptic forms of long-term plasticity, postsynaptic calcium influx and ionotropic glutamate receptor activity (NMDA- and AMPA-type receptors) are not required (Harris and Cotman, 1986; Zalutsky and Nicoll, 1990; Castillo et al., 1994; Langdon et al., 1995; Kobayashi et al., 1996; Tzounopoulos et al., 1998; Kobayashi et al., 1999; Mellor and Nicoll, 2001).

Besides the presynaptically induced and expressed form of plasticity described above, the mossy fiber-CA3 synapses have also been reported to undergo spike-timing-dependent LTP and LTP selectively expressed by NMDA receptors; both forms of LTP require postsynaptic calcium rise during induction (Jaffe and Johnston, 1990; Urban and Barrionuevo, 1996; Kapur et al., 1998; Sokolov et al., 2003; Kwon and Castillo, 2008; Rebola et al., 2008). After potentiation of NMDA receptors, these synapse become capable of expressing NMDAR-dependent LTP of AMPA receptors (Rebola et al., 2011). Recently, it has been shown that a postsynaptic form of LTP can also be unmasked when synaptic zinc is chelated (Pan et al., 2011). The detailed mechanisms for these LTP are beyond the scope of the current review, which focuses on presynaptic forms of long-term plasticity. However, these observations demonstrate that even within a given synapse type, experimental conditions and induction protocols may elicit mechanistically distinct forms of long-term plasticity—such findings have been recapitulated in diverse brain circuits.

#### LTP at cerebellar parallel fiber-Purkinje cell synapses

Cerebellar parallel fibers are the axon tracts from cerebellar granule cells that synapse onto cerebellar Purkinje cells. The strength of these synapses is dynamically regulated, and multiple forms of plasticity are required for motor learning (Boyden et al., 2006). The parallel fiber-Purkinje synapses exhibit a presynaptic LTP similar to that of the hippocampal mossy fiber-CA3 synapses (Salin et al., 1996; Castillo et al., 2002). This presynaptic LTP is expressed by an increase in neurotransmitter release, as evidenced by a reduction in synaptic failure rates and a decrease in PPR (Salin et al., 1996; Bender et al., 2009). The increase in neurotransmitter release is associated with an increase in quantal content, potentially indicating a shift from single to multi-vesicular release (Bender et al., 2009). Parallel fiber LTP is induced presynaptically; it is resistant to glutamate receptor antagonists and calcium chelation at postsynaptic Purkinje cells, but requires presynaptic calcium influx (Salin et al., 1996).

#### LTP at cortico-thalamic synapses

The thalamus and neocortex are two highly organized and complex brain structures that work in concert with each other. A majority of afferents to the neocortex originates in the thalamus, and in return, the neocortex has massive connectivity to the thalamus through cortico-thalamic synapses. This loop is critical for motor function and is also considered as an important means for cortico-cortical communication (Guillery and Sherman, 2002). At the glutamatergic synapses between cortico-thalamic fibers and the ventrobasal thalamus, LTP can be induced and expressed presynaptically. This presynaptic LTP has been shown to be associated with a decrease in PPR and is resistant to glutamate receptor antagonists (Castro-Alamancos and Calcagnotto, 1999).

#### LTP at synapses between cortical fast-spiking interneurons

Fast-spiking interneurons (FSI) are implicated in synchronizing activity among projection neurons and establishing gamma oscillations (Bartos et al., 2007; and Fries et al., 2007; Humphries et al., 2009). FSI dysfunction is implicated in diseases such as schizophrenia and Dravet Syndrome (Yamakawa, 2011; Nakazawa et al., 2012). A presynaptic form of LTP has recently been described in FSIs at layer II/III of the mouse visual cortex (Sarihi et al., 2012). Tetanic stimulation of the presynaptic FSI in a paired whole-cell recording configuration demonstrated potentiation of unitary IPSCs in FSI → FSI pairs, but not non-FSI → FSI pairs. A similar potentiation of FSI IPSPs was observed by tetanic stimulation of nearby afferents under conditions blocking glutamatergic transmission. This IPSP potentiation was associated with concomitant changes in PPR and *CV*^2^ to support a presynaptic mechanism. In addition, this form of FSI-FSI LTP was insensitive to manipulations that blocked rises in postsynaptic calcium or inhibited activity of mGluRs or L- or T-type voltage-gated calcium channels, suggesting that induction may also depend solely on presynaptic activity (Sarihi et al., 2012). Because LTP of an inhibitory synapse onto an FSI would result in long-lasting suppression of FS interneurons, this form of presynaptic LTP between FS interneurons may function to desynchronize the cortical network.

#### Presynaptic mGluR2/3-mediated LTD

As mentioned earlier, activation of presynaptic mGluR2/3 is involved in LTD at the hippocampal mossy fiber-CA3 synapses (Tzounopoulos et al., 1998). This involvement is also observed in LTD at nucleus accumbens synapses (Robbe et al., 2002a). Bath application of an mGluR2/3 specific agonist, L-CCG-1 or LY354740, depresses synaptic transmission and occludes further tetanus-induced LTD at both synapses, suggesting that activation of type II mGluRs at the presynaptic terminals is sufficient for the induction of LTD (Tzounopoulos et al., 1998; Robbe et al., 2002b). Evidence for presynaptic expression for this form of LTD includes observations of an increased PPR and a decreased miniature EPSC frequency at both synapses (Tzounopoulos et al., 1998; Robbe et al., 2002a).

### Postsynaptically induced and presynaptically expressed long-term plasticity

#### Endocannabinoid-mediated LTD

Retrograde signaling can be achieved by the diffusion of small molecules released from postsynaptic spines. One such example is through endocannabinoids (eCBs). Retrograde signaling by eCBs has become a heavily studied topic over the past decade and multiple excellent reviews have covered various aspects of this signaling pathway (Alger, 2002; Freund et al., 2003; Gerdeman and Lovinger, 2003; Chevaleyre et al., 2006; Lovinger, 2008; Heifets and Castillo, 2009; Kano et al., 2009). Endocannabinoid-mediated presynaptic LTD has been reported in many brain regions, such as the striatum (Gerdeman et al., 2002; Kreitzer and Malenka, 2005; Shen et al., 2008), the nucleus accumbens (Robbe et al., 2002b; Mato et al., 2008), amygdala (Marsicano et al., 2002; Huang et al., 2003; Azad et al., 2004), hippocampus (Chevaleyre and Castillo, 2003; Chevaleyre et al., 2007; Yasuda et al., 2008), cerebellum (Soler-Llavina and Sabatini, 2006), visual cortex (Sjöström et al., 2003, 2004; Crozier et al., 2007; Huang et al., 2008a), somatosensory cortex (Bender et al., 2006; Li et al., 2009), prefrontal cortex (Lafourcade et al., 2007), VTA (Pan et al., 2008; Haj-Dahmane and Shen, 2010), hypothalamus (Kuzmiski et al., 2009; Crosby et al., 2011), brain stem (Tzounopoulos et al., 2007) and superior colliculus (Henneberger et al., 2007).

In general, eCB-LTD requires the production of eCB from the postsynaptic neuron. However, the exact induction mechanisms differ widely across examples of eCB-LTD. These differences include different requirements of postsynaptic receptors or kinases to stimulate eCB production, as well as differences in the stimulation pattern required to induce LTD. For a detailed description of the induction mechanism in various synapses, please refer to two excellent reviews on the topic (Gerdeman and Lovinger, 2003; Heifets and Castillo, 2009).

Upon activation of the CB1R at presynaptic terminals, eCB-LTD is expressed as an overall decrease in neurotransmitter release, indicated by decreased frequency of quantal synaptic events (Robbe et al., 2002b; Huang et al., 2003; Sjöström et al., 2003; Bender et al., 2006; Lafourcade et al., 2007; Pan et al., 2008), increased PPR (Gerdeman et al., 2002; Chevaleyre and Castillo, 2003; Huang et al., 2003; Soler-Llavina and Sabatini, 2006; Tzounopoulos et al., 2007; Pan et al., 2008; Kuzmiski et al., 2009; Haj-Dahmane and Shen, 2010), increased *CV*^2^ (Sjöström et al., 2003; Tzounopoulos et al., 2007; Pan et al., 2008; Yasuda et al., 2008; Kuzmiski et al., 2009), and increased failure rate upon minimal stimulation (Soler-Llavina and Sabatini, 2006; Kuzmiski et al., 2009; Haj-Dahmane and Shen, 2010).

Not all forms of eCB-LTD are homosynaptic. Endocannabinoid-mediated heterosynaptic LTD is observed at GABAergic synapses in hippocampus, striatum, frontal cortex, VTA and superior colliculus, and is thought to arise from eCB spillover from nearby glutamatergic synapses expressing homosynaptic LTD (Chevaleyre and Castillo, 2003; Henneberger et al., 2007; Pan et al., 2008; Adermark et al., 2009; Chiu et al., 2010). Heterosynaptic eCB-LTD is also observed at glutamatergic synapses between layer 4 and layer 2/3 neurons in visual cortex of young mice (Huang et al., 2008a). These heterosynaptic forms of eCB-LTD do not require the activation of presynaptic neurons during induction, thus, not following the Hebbian rule. Both homo- and heterosynaptic eCB-LTD are important for normal brain functions and behaviors, and are implicated in disease models (Marsicano et al., 2002; Crozier et al., 2007; Huang et al., 2008a; Kuzmiski et al., 2009; Li et al., 2009; Lerner et al., 2010; Zhang and Alger, 2010).

#### Nitric oxide (NO)-dependent presynaptic long-term plasticity

A second example of retrograde signaling is nitric oxide. Nitric oxide is a small diffusible molecule with a short lifetime, and is generated by nitric oxide synthase (NOS) (Alderton et al., 2001). In neurons, NOS is coupled to NMDA receptors, and activated by Ca^2+^/calmodulin upon the opening of NMDA receptors (Marin et al., 1992). Due to its high mobility and ability to cross membranes, NO can act locally at the post-synaptic neuron, as an anterograde messeger, a retrograde messeger, or a volume messenger to nearby cells without synaptic activity or NOS expression (Arancio et al., 1996; Lev-Ram et al., 1997; Park et al., 1998; Ko and Kelly, 1999; Ledo et al., 2005; Garthwaite, 2008; Steinert et al., 2010). As a retrograde messeger, NO has been shown to reduce glutamate release at excitatory synapses (Gage et al., 1997; Stanton et al., 2003) and to increase GABA release at inhibitory synapses (Bains and Ferguson, 1997; Stern and Ludwig, 2001).

NO has been shown to play a role in presynaptic long-term plasticity at various synapses. It is required for a form of NMDA receptor-dependent presynaptic LTD at hippocampal CA3-CA1 synapses, and thought to serve as a Hebbian coincidence detector for the activation of both presynaptic and postsynaptic neurons (Stanton et al., 2003; Zhang et al., 2006). It also acts heterosynaptically to support presynaptic LTP at GABAergic synapses onto dopamine neurons in VTA (Nugent et al., 2007). Further, it is reported that NO is required heterosynaptically for presynaptic LTP and eCB-mediated presynaptic LTD at GABAergic synapses in the dorsomedial hypothalamus, suggesting a state-dependent action of NO and crosstalk between various signaling pathways [Crosby et al., 2011; will be discussed in State-Dependent LTP at Inhibitory Synapses in the Dorsomedial Nucleus of the Hypothalamus (DMH)].

Interestingly, although the heterosynaptic forms of NO-LTP at inhibitory synapses require activation of NMDA-R of the postsynaptic neurons (Nugent et al., 2007; Crosby et al., 2011), they do not require activity of the presynaptic neuron during induction (Nugent et al., 2007). These non-Hebbian forms of LTP may serve to maintain excitatory/inhibitory balance and circuit homeostasis, up-regulating the local inhibitory tone in response to elevated excitatory inputs.

#### LTP and LTD at hippocampal CA3-CA1 synapses

The hippocampal CA3-CA1 Schaffer collateral-commissural synapse displays multiple forms of long-term plasticity. At this synapse, LTP can be readily induced in juvenile and adult animals with a variety of induction protocols, such as 50, 100, and 200 Hz train stimulation. LTP in adult animals induced with a single 50 or 100 Hz tetanus requires activation of NMDA receptors and postsynaptic calcium influx, and is expressed primarily by an addition of surface AMPA receptors to the postsynaptic spines (Nicoll and Malenka, 1999b; Malinow et al., 2000). However, other groups have found that LTP induced with a 200 Hz (or multiple trains of 100 Hz) tetanus or specific presynaptic and postsynaptic pairing protocols may involve an additional presynaptic component besides postsynaptic LTP of AMPA receptor insertion (Zakharenko et al., 2002; Emptage et al., 2003; Bayazitov et al., 2007; Enoki et al., 2009). This presynaptic component is suggested by a decrease in *CV*, PPR and minimal stimulation failure rate (Malinow, 1991; Sokolov et al., 2002; Emptage et al., 2003; but see Nicoll and Malenka, 1999b), and supported by evidence at the single synapse level of an increase in the number of post-synapses showing EPSCaT signal (Emptage et al., 2003; Enoki et al., 2009), an accelerated rate of FM dye destaining (Zakharenko et al., 2002, 2003), and an enhanced synapto-pHluorin signal (Bayazitov et al., 2007) after LTP. The induction of this presynaptic component requires postsynaptic depolarization and subsequent calcium influx through L-type calcium channels (Zakharenko et al., 2003; Bayazitov et al., 2007), yet the exact retrograde signal for this presynaptic component remains to be investigated. A presynaptically expressed CA3-CA1 LTP has also been described in neonatal animals (P6 rats) with presynaptic and postsynaptic pairing protocols; this LTP is expressed in presynaptic terminals with low initial release probability and is associated with a decrease in failure rate and PPR (Palmer et al., 2004).

Besides LTP, CA3-CA1 synapse can also undergo LTD with both presynaptic and postsynaptic expression, for example, in cases of mGluR-LTD. Postsynaptic activation of group 1 mGluRs, either by its selective agonist DHPG or by appropriate synaptic stimulation, leads to postsynaptic and presynaptic changes that reduce the efficacy of the CA3-CA1 synapses—a rapid removal of surface AMPARs postsynaptically (Snyder et al., 2001; Xiao et al., 2001), and a reduction in presynaptic vesicle release suggested by a slower rate of FM dye destaining, an increase in failure rate and an increase in both *CV*^2^ and PPR (Fitzjohn et al., 2001; Faas et al., 2002; Zakharenko et al., 2002). A slower rate of FM dye destaining and an increase in PPR also accompany a form of LTD induced by prolonged activation of NMDA receptors (Stanton et al., 2003; Zhang et al., 2006). It has been shown that bath application of selective NOS inhibitor L-nitroarginine partially reduced the degree of depression but completely reversed the change of FM dye destaining, suggesting that this LTD has postsynaptic and presynaptic components, with the presynaptic expression dependent on nitric oxide signaling (Stanton et al., 2003). It suggested that NO works as a retrograde messenger from activation of postsynaptic NMDA receptors because the presynaptic depression can be partially blocked by extracellular NO scavenger (Stanton et al., 2003). A third example of presynaptic LTD at CA3-CA1 synapses is induced by a specific presynaptic and postsynaptic pairing protocol and associated with a decrease in the number of post-synapses showing EPSCaT signal (Enoki et al., 2009).

These Hebbian forms of compound long-term plasticity ensure effective modulation of synaptic strength - the degree of modulation is magnified when both pre- and post-synaptic potentiation (or depression) occur together.

### Presynaptic NMDAR-dependent homo- and heterosynaptic long-term plasticity

Once again, not all forms of synaptic plasticity are homo-synaptic. Heterosynaptic long-term plasticity, the induction of which requires activity of other synapses, may result from diffusible molecules binding to receptors at the presynaptic terminal. These diffusible molecules can be released from postsynaptic neurons, as with eCBs; but they can also arise from nearby presynaptic terminals, as in presynaptic NMDAR dependent LTP induced by glutamate. Heterosynaptic NMDAR-dependent LTP at excitatory neurons can serve as a coincidence detector for two different inputs, altering their relative strength and how the postsynaptic neurons integrate information from different sources. Heterosynaptic NMDAR-dependent LTP at inhibitory neurons may be involved in regulation of excitability and shifting the circuit balance for excitatory and inhibitory inputs.

#### LTP at cortical afferents to the lateral amygdala

Lateral amygdala (LA) receives input from both the cortex and thalamus, and is involved in mediating emotional responses, especially fear-associated learning and memory (LeDoux, 2000). Cortical afferents to the LA can undergo both homosynaptic and heterosynaptic LTP. Homosynaptic LTP can be induced with various protocols; both a Hebbian form and a non-Hebbian form have been described. The Hebbian form requires calcium influx through L-type calcium channel at the postsynaptic terminals and activation of NMDA receptors (Huang and Kandel, 1998; Tsvetkov et al., 2002), while the non-Hebbian form requires no ionotropic glutamatergic transmission but calcium influx into the presynaptic terminals and blockage of presynaptic GABA_B_ receptors (Shaban et al., 2006). Heterosynaptic LTP requires activation of presynaptic NMDA receptors and presynaptic calcium influx in the cortical afferents at the same time, which is achieved only with simultaneous activation of both cortical and thalamic afferents, or upon a puff-application of NMDA coincident with train stimulation of cortical afferents (Humeau et al., 2003). Though induced differently, homosynaptic and heterosynaptic forms of LTP occlude each other, and are expressed presynaptically as an increase in neurotransmitter release, as evidenced by a decrease in PPR, *CV*^2^, a reduction in failure rate under minimal stimulation, and a faster MK801 blockade of NMDAR-mediating currents (Huang and Kandel, 1998; Tsvetkov et al., 2002; Humeau et al., 2003; Shaban et al., 2006; Fourcaudot et al., 2008). Both forms of LTP and corresponding presynaptic changes are occluded after fear conditioning, suggesting that presynaptically expressed LTP at the cortico-LA synapses may underlie fear memory (Tsvetkov et al., 2002; Shaban et al., 2006).

#### LTP at cerebellar stellate cell-stellate cell synapses

In the cerebellum, stellate cells form inhibitory synapses onto Purkinje cells, regulating synaptic integration properties as well as the timing and firing frequencies of Purkinje cells (Häusser and Clark, 1997). Stellate cells are also connected to each other via inhibitory synapses and form an inhibitory network (Mann-Metzer and Yarom, 1999). A heterosynaptic form of LTP has recently been reported at the stellate cell-stellate cell synapses (Lachamp et al., 2009). At these inhibitory synapses, NMDA receptors are present at presynaptic terminals and can be activated by glutamate released from parallel fibers (Glitsch and Marty, 1999; Liu and Lachamp, 2006). Activation of presynaptic NMDA receptors by tetanus of the parallel fibers or by brief application of NMDA greatly potentiates transmission at these synapses. This potentiation is due to an increase in GABA release, as evidenced by a reduction in PPR, *CV*^2^, failure rate under minimal stimulation, and an increased frequency of miniature IPSCs (Lachamp et al., 2009). Importantly, this form of LTP can be induced without firing of the presynaptic stellate cell (Lachamp et al., 2009). This type of presynaptic LTP might provide a mechanism for the inhibitory network to sense and balance excessive excitation in the circuit.

### State dependent presynaptic long-term plasticity

It is well known that synapses are capable of expressing multiple forms of long-term plasticity. Why might there be multiple forms, and how might the synapse regulate which are used? Behaviorally, depending on past experience and the internal state of an animal, we know that the same stimulus can evoke different responses. Recent studies have found that this is also true at the synaptic level. Here we review three examples of how changes in presynaptic surface receptors can gate the ability of a synapse to express certain forms of long-term plasticity, such that the same induction protocol elicits different plasticity. This state-dependent plasticity may underlie hierarchical information processing and is important for proper behavioral responses.

#### LTD and de-depresssion at hippocampal mossy fiber-interneuron synapses

Besides CA3 pyramidal neurons, mossy fibers also synapse onto CA3 stratum lucidum interneurons (SLIN). Unlike mossy fiber-CA3 synapses that are large (~3–8 μm in diameter) with multiple release sites (Rollenhagen and Lübke, 2010), mossy fiber-SLIN synapses are small terminations (~1 μm) of numerous fine filopodia that radiate from mossy fiber boutons (Acsády et al., 1998). After HFS of mossy fiber inputs, which produces presynaptic LTP at the CA3 pyramidal synapses, those SLINs which contain calcium-permeable AMPA receptors will exhibit LTD presynaptically (Maccaferri et al., 1998; Lei and McBain, 2004; Pelkey et al., 2005). This presynaptically expressed LTD follows a Hebbian learning rule, as it requires calcium influx through postsynaptic AMPA receptors and activation of presynaptic mGluR7b receptors (Laezza et al., 1999; Pelkey et al., 2005). Upon activation, presynaptic mGluR7bs are internalized. Without surface mGluR7b, the same synapses will now exhibit de-depression with a second train of HFS. Although not a mechanistic reversal of LTD, de-depression is induced presynaptically and expressed as an increase in neurotransmitter release (Pelkey et al., 2005; Pelkey and McBain, 2008). The ability of the mossy fiber-SLIN filopodia synapses to undergo presynaptic LTD and meta-plasticity may allow for profound and rapid state-dependent control of feed-forward inhibition in the hippocampal network, and is implicated in spatial and temporal information processing (Kullmann and Lamsa, 2008; McBain and Kauer, 2009).

A second class of presynaptic receptors gating the polarity of presynaptic long-term plasticity is CB1 receptors. Activation of CB1 receptors by eCBs has been shown to prevent the expression of presynaptic LTP at multiple synapses with behavioral significance, and is discussed in the following 2 subsections.

#### LTP at thalamic afferents to the lateral amygdala

LTP of thalamic synapses onto LA has generally been found to be expressed postsynaptically through changes in AMPA receptor trafficking (Humeau et al., 2005; Rumpel et al., 2005). One recent study, however, provides evidence for conditions under which thalamic-LA synapses may also undergo presynaptic LTP, which is gated by CB1 receptor activation (Shin et al., 2010b). When postsynaptic LA neurons were held at a hyperpolarized potential (−70 mV), repetitive stimulation of thalamic afferents resulted in presynaptic LTP, as evidenced by a reduction in PPR as well as failure rate under minimal stimulation. This LTP was induced presynaptically; it required activation of presynaptic kainate receptors (KAR) and calcium influx into the presynaptic terminals, but not NMDAR activation nor calcium influx into the postsynaptic LA neurons. Interestingly, when postsynaptic LA neurons were held at depolarized potential (+30 mV) during induction, the same presynaptic stimulation for KAR-dependent presynaptic LTP would instead lead to postsynaptic LTP and suppression of presynaptic LTP. The suppression of presynaptic LTP appears to be mediated by activation of presynaptic CB1Rs. When CB1Rs are blocked, the presynaptic LTP is unmasked and can be expressed simultaneously with postsynaptic LTP at these synapses (Shin et al., 2010b).

These findings suggest that the eCB system is employed to report activation of postsynaptic LA neurons and prevent over potentiation of the thalamic afferents. When thalamic input fails to depolarize postsynaptic LA neurons, presynaptic LTP is employed to ensure efficient synaptic transmission. However, because glutamate released from thalamic afferents can activate presynaptic NMDA receptor to promote heterosynaptic LTP at cortico-LA synapses (Humeau et al., 2003), unchecked potentiation of the thalamic afferents may lead to inappropriate potentiation of cortico-LA synapses during fear conditioning, potentially resulting in increased fear experience and fear generalization. Gating of presynaptic LTP at thalamic-LA synapses by eCB and CB1R may thus serve as an activity-dependent constraint to prevent the generalization of conditioned fear. Indeed, behavioral studies have shown that CB1R antagonism enhanced baseline freezing, which is usually attributed to fear generalization (Reich et al., 2008).

#### State-dependent LTP at inhibitory synapses in the dorsomedial nucleus of the hypothalamus (DMH)

Studies of the DMH provide some of the first direct evidence that the internal state of an animal can alter presynaptic surface receptors and gate presynaptic plasticity. The DMH has roles in signaling satiety and regulating food intake (Bellinger and Bernardis, 2002). Recent work provides evidence that the feeding state of an animal reduces CB1R signaling and determines the polarity of long-term plasticity in inhibitory synapses in DMH (Crosby et al., 2011). When mice are given *ad libitum* access to food, repeated stimulus trains (100 Hz, 4 s) induces presynaptic LTD at inhibitory synaspes in DMH, as supported by an increase in PPR and *CV*. This LTD requires activation of presynaptic CB1R by eCBs and is dependent on NO production. Both eCBs and NO are thought to be produced heterosynaptically by excitatory synapses. When mice were food deprived, however, the same tetanus produces presynaptic LTP at these synapses, which is dependent on the production of NO (Crosby et al., 2011). It was further shown that food deprivation dampens CB1R signaling at inhibitory synapses in DMH and blockade of CB1R in naïve mice shifts presynaptic LTD to LTP similar to that in food deprivation, suggesting that food availability is encoded at these synapses by presynaptic CB1Rs which gates their ability of to undergo LTP. Because DMH neurons are thought to send excitatory projections to the paraventral nucleus of the hypothalamus (PVN) that suppresses food intake, an increase in inhibitory drive in DMH would suppress neuronal activities in PVN, ultimately leading to an elevated drive to eat when food is limited (Crosby et al., 2011).

CB1 receptors are widely expressed in the brain and highly regulated by neuronal activities and behavioral states of an animal. It is possible that presynaptic CB1R may affect the expression of different forms of long-term plasticity in other brain regions besides mediating eCB-LTD. Other receptors at the presynaptic terminals, such as NMDA receptors, kainate receptors, mGluRs and GABA_B_ receptors, can also be regulated by activity and influence the plasticity decision of a synapse. As such, gating of synaptic plasticity by presynaptic surface receptors may turn out to be a more widespread phenomenon.

## Molecular mechanisms

The molecular mechanisms underlying presynaptically expressed long-term plasticity and their maintenance have been a focus of the field for decades. It was recognized early on that the cyclic adenosine monophosphate (cAMP) and cAMP-dependent protein kinase A (PKA) pathway is required for mossy fiber LTP in guinea pigs (Weisskopf et al., 1994), rats (Huang et al., 1994) and mice (Huang et al., 1995). Here, we review evidence for the involvement of the cAMP/PKA pathway for various forms of presynaptically expressed LTP and the notion that a shift in the balance of kinase and phosphatase activity underlies presynaptically expressed LTD. We then review what processes downstream of, or in parallel with, these signaling pathways can account for the long-term alteration of neurotransmitter release. Changes in neurotransmitter release efficacy can theoretically result from diverse mechanisms, such as modifications of the release machinery, alterations in calcium influx through voltage-gated calcium channels, or changes in intrinsic membrane excitability of the presynaptic terminal.

### cAMP/PKA cascade in presynaptically expressed long-term plasticity

#### cAMP/PKA cascade in presynaptically expressed LTP

The first evidence implicating cAMP/PKA signaling in presynaptic LTP was observed at hippocampal mossy fiber-CA3 synapses. In guinea pigs, the Nicoll group (Weisskopf et al., 1994) discovered that brief application of the adenylyl cyclase activator, forskolin, increased basal transmission at mossy fiber synapses in an enduring manner, and occluded electrically stimulated presynaptic LTP. Sp-cAMPs, analogs of cAMP that activate PKA more selectively, also enhanced basal transmission long-lastingly following brief application, and this effect was occluded by prior application of forskolin. These findings indicated that brief elevation of PKA activity might be sufficient for mossy fiber LTP. Weisskopf and colleagues further established the necessity of the cAMP/PKA cascade by using three different PKA inhibitors (Weisskopf et al., 1994). Blockers of the catalytic subunit of PKA, KT5720 and H-89, as well as an inhibitor of the regulatory subunit, Rp-cAMPs, all antagonized mossy fiber LTP, without any effect on basal transmission. These effects were localized to the presynaptic terminal by demonstrating that a reduction of PPR accompanied forskolin-enhanced transmission and that postsynaptic responses to iontophoretic pulse of glutamate remained constant. Together, these results suggested that a brief elevation of cAMP causes persistent increase in neurotransmitter release and that PKA activation is an essential step in mossy fiber LTP (Weisskopf et al., 1994). Similar results were observed at hippocampal mossy fiber-CA3 synapses in rats and mice (Huang et al., 1994; Spillane et al., 1995; Castillo et al., 1997).

The cAMP/PKA pathway has subsequently been shown to be involved in presynaptically expressed LTP in many brain regions. Forskolin enhances basal neurotransmitter release and occludes LTP at cerebellar parallel fiber-Purkinje cell synapses (Salin et al., 1996), cortico-thalamic synapses (Domenici et al., 1998), the cortical afferents to LA (Huang and Kandel, 1998), as well as inhibitory synapses between cerebellar stellate cells (Lachamp et al., 2009). Similarly, LTP at these synapses is blocked by the PKA inhibitor, Rp-cAMPs (Salin et al., 1996; Domenici et al., 1998; Huang and Kandel, 1998; Lachamp et al., 2009). These results support that cAMP/PKA activation underlies presynaptically expressed LTP. Interestingly, the cAMP/PKA pathway also functions at the hippocampal mossy fiber-interneuron synapses, but only under certain conditions. As reviewed earlier, these synapses undergo presynaptic LTD when presynaptic mGluR7b receptors are at the surface, but exhibit de-depression once those receptors are internalized (Pelkey et al., 2008). Besides gating the direction of presynaptic plasticity, surface mGluR7b also gates the sensitivity to cAMP and PKA. When mGluR7b are at the surface, forskolin has no effect on basal transmission. Only after mGluR7b are internalized, does forskolin enhance basal transmission (Pelkey et al., 2008). While the adenylyl cyclase inhibitor, DDOA, or the PKA inhibitor, H-89, did not affect mGluR7b mediated presynaptic depression, both drugs abolished subsequent de-depression after mGluR7b internalization. In all, these results showed that, following internalization of presynaptic surface mGluR7b receptors, the cAMP/PKA pathway is involved in presynaptic de-depression at the hippocampal mossy fiber-interneuron synapses (Pelkey et al., 2008).

One note about the forskolin effect of enhancing transmission is warranted. Although this phenomenon completely occluded subsequent tetanus-induced LTP, tetanus-induced LTP only partially reduced the enhancement by brief application of forskolin (Weisskopf et al., 1994). This observation can be readily explained if there are multiple degrees of potentiation and/or heterogeneous induction thresholds for different afferents, such that not all electrically tetanized synapses are potentiated to their maximum. Alternatively, forskolin may activate multiple mechanisms to enhance neurotransmitter release (Lonart et al., 1998), some of which may not normally be engaged by tetanus, the cAMP/PKA pathway, or under physiological conditions. Therefore, one has to be cautious when comparing pharmacological- vs. activity-induced long-term plasticity results.

A requirement for the cAMP/PKA pathway in presynaptic LTP has also been shown genetically in studies using mice lacking the genes encoding adenylyl cyclase or PKA. In the type-1 adenylyl cyclase knockout mouse, hippocampal mossy fiber LTP was selectively impaired, leaving the prefrontal pathway LTP and the Schaffer collateral LTP intact (Villacres et al., 1998). In this mouse, high concentrations of forskolin were still capable of enhancing transmission, suggesting that downstream signaling of cAMP was intact. In the PKA knockout mouse, both electrically stimulated mossy fiber LTP and forskolin enhanced synaptic transmission were abolished, confirming the essential role of cAMP/PKA signaling in presynaptically expressed LTP (Huang et al., 1995).

#### Reduction of cAMP/PKA activity in presynaptically expressed LTD

Cyclic AMP/PKA activity is thought to be down-regulated in presynaptically expressed LTD at hippocampal mossy fiber-CA3 synapses (Tzounopoulos et al., 1998), hippocampal interneuron-pyramidal cell synapses (Chevaleyre et al., 2007), nucleus accumbens synapses (Robbe et al., 2002a; Mato et al., 2008), and excitatory synapses onto dopamine neurons in the VTA (Haj-Dahmane and Shen, 2010). PKA inhibitors, KT5720, H-89 or PKI have been shown to occlude tetanus-induced or pharmacologically-induced LTD by suppressing basal transmission at the nucleus accumbens synapses and the hippocampal interneuron-pyramidal cell synapses (Robbe et al., 2002a; Chevaleyre et al., 2007; Mato et al., 2008). The down-regulation of PKA is localized to the presynaptic neuron by the findings that neither basal transmission nor LTD was altered when a membrane impermeable form of PKI was delivered to the postsynaptic cell via the recording pipette (Chevaleyre et al., 2007).

Interestingly, presynaptic LTD at these three synapses requires activation of G-protein coupled receptors, mGluR2/3s or CB1Rs. At the nucleus accumbens synapses, where both receptors are present at the presynaptic terminals, mGluR2/3-LTD and eCB-LTD mutually occlude, suggesting that both forms of presynaptic LTD share a common mechanism (Mato et al., 2005). Indeed, both receptors are coupled to Gα_*i*_. The α_*i*_ effector limb shared by these two receptor types would inhibit adenylyl cyclase activity and thereby the cAMP/PKA pathway (Pin and Duvoisin, 1995; Childers and Deadwyler, 1996). The involvement of Gα_*i*_-coupled receptors and their known inhibition of adenylyl cyclase further suggest that presynaptic LTD may be expressed through a reduction in cAMP/PKA kinase activity. Importantly, brief activation of CB1R alone is not sufficient to induce presynaptic LTD, suggesting that other signaling pathways might be involved besides a reduction in cAMP/PKA kinase activity (Gerdeman et al., 2002; Heifets et al., 2008; Heifets and Castillo, 2009; Lovinger, 2010). At hippocampal interneuron-pyramidal cell synapses, Heifets et al. (2008) found that, calcineurin, a calcium-activated phosphatase, is required for eCB-LTD. This finding supports a model whereby the balance of kinase and phosphatase activity is shifted in presynaptically expressed long-term plasticity to favor dephosphorylation in LTD.

The involvement of the cAMP/PKA pathway in presynaptically expressed long-term plasticity has been firmly established in the last decades. However, the downstream effector has not yet been fully identified. Two presynaptic proteins, synapsin and rabphilin, that are both phosphorylated by PKA are not required for presynaptic long-term plasticity (Spillane et al., 1995; Lonart and Südhof, 1998; Hosaka et al., 1999; Schlüter et al., 1999). Another PKA substrate at the presynaptic terminal, RIM1α, is required for many forms of presynaptic long-term plasticity (reviewed in the next section). However, in the RIM1α knockout mouse, despite the lack of mossy fiber LTP, basal transmission can still be enhanced by forskolin, suggesting that PKA may function in parallel with RIM1α (Castillo et al., 2002). Furthermore, the significance of a PKA phosphorylation site on RIM1α, Serine 413, although shown to be necessary for presynaptic LTP in a cerebellar culture model (Lonart et al., 2003; Simsek-Duran et al., 2004), was not supported when presynaptic LTP was studied in more *in vivo* brain preparations. Using acute brain slices from a genetic knockin model (S413A) and in an acute *in vivo* rescue approach, mutation of the S413 site did not affect expression of presynaptic LTP (Kaeser et al., 2008b; Yang and Calakos, 2010). Therefore, the PKA targets underlying presynaptic LTP remain to be elucidated.

### Modifiers of synaptic release probability

**Figure 2:**
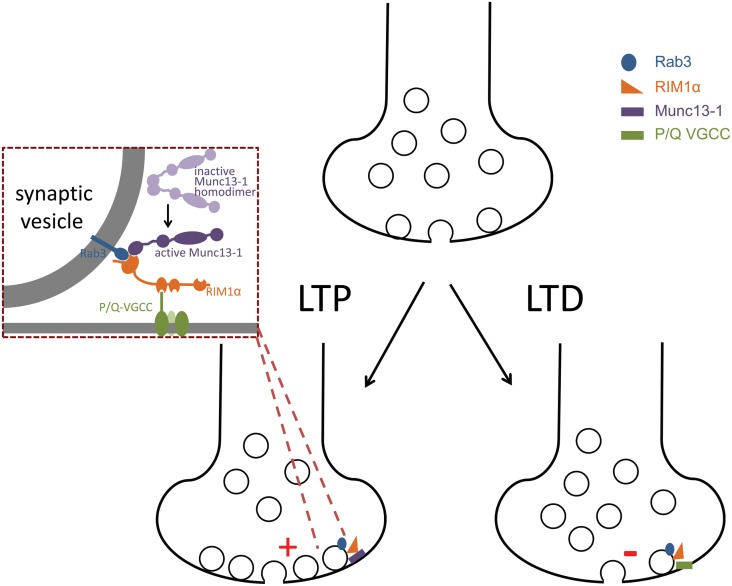
**Working models of expression mechanisms for presynaptic LTP and LTD**. In this schematic, presynaptic molecules implicated in the expression of changes in presynaptic release probability associated with LTP and LTD are shown. In LTP at mossy fiber synapses, the specific interaction of RIM1a with Munc13-1 that promotes activation of Munc13 for vesicle priming is required, indicating that a likely cellular mechanism for increasing release probability may be through changes in the number of primed vesicles (indicated by vesicles adjacent to the terminal membrane). In LTD at hippocampal mossy fiber—stratum lucidum interneuron synapses and the nucleus accumbens, inhibition of P/Q type voltage-gated calcium channel (VGCC) activity is associated with expression of LTD. Whether this modulation of channel activity requires interaction with RIM1a has not been tested. At other synapses, a requirement for Rab3 and RIM1a for LTD has been described. Therefore, in the LTD working model, we also hypothesize that LTD mechanisms opposite to LTP may occur and thus show fewer primed vesicles to lead to a decrease in release probability. However, a requirement for Munc13 in LTD has not yet been tested. In addition, while Rab3a and RIM1a are both implicated in LTP and LTD, whether their interaction is required has also not been tested. Lastly, whether multiple expression mechanisms co-exist within a synapse or occur at distinct subsets of synapses remains to be determined.

Vesicle exocytosis is tightly regulated at the presynaptic terminal and a major determinant of synaptic release probability. In this section, we will discuss molecular targets for presynaptic long-term plasticity that affect synaptic vesicle release efficacy (Figure 2).

#### Rim1α

RIM1α is a presynaptic scaffolding protein of the active zone; it binds to many molecules involved in neurotransmitter release, such as Munc13 (Betz et al., 2001; Schoch et al., 2002; Deng et al., 2011), Synaptotagmin1 (Coppola et al., 2001; Schoch et al., 2002), Rab3 (Wang et al., 1997; Schoch et al., 2002) and voltage-gated calcium channles (VGCCs) (Coppola et al., 2001; Wang et al., 2001; Kiyonaka et al., 2007; Kaeser et al., 2011; Han et al., 2011). RIM1α is required for presynaptic LTP at hippocampal mossy fiber-CA3 synapses, Schaffer collateral-CA1 synapses, cerebellar parallel fiber synapses, cortical LA synapses, as well as presynaptic LTD at inhibitory synapses in the hippocampus, in basolateral amygdala, and at cerebellar stellate cell synapses (Castillo et al., 2002; Huang et al., 2005; Chevaleyre et al., 2007; Fourcaudot et al., 2008; Lachamp et al., 2009). Interestingly, while deleting RIM1α abolishes presynaptic LTD at inhibitory synapses, RIM1α deletion enhances presynaptic LTD at hippocampal mossy fiber-CA3 synapses (Castillo et al., 2002). These observations indicate that RIM1α may have different functions at excitatory and inhibitory synapses. Nevertheless, changes in the release machinery via RIM1α may be a general mechanism underlying presynaptic long-term plasticity. RIM1α has been shown to enhance neurotransmission by promoting vesicle priming, a process by which vesicles at the active zone become fusion competent (Calakos et al., 2004). The ability of RIM1α to promote vesicle priming is mediated through its interaction with the priming protein, Munc13 (Betz et al., 2001; Deng et al., 2011). Of the myriad potential protein interactions and synaptic modifications that RIM1a may influence, we recently showed that the interaction of RIM1α and Munc13 required to modulate vesicle priming is also required for presynaptic LTP at hippocampal mossy fiber synapses (Yang and Calakos, 2011). These data indicate that regulation of vesicle priming via a RIM-Munc13 interaction is a likely cellular substrate for enhancement of presynaptic strength in presynaptic LTP.

#### Rab3

Rab3 proteins (Rab3A/B/C/D) are a family of small GTPases located on synaptic vesicles. They cycle between the GDP-bound inactive form and GTP-bound active form and are associated with synaptic vesicle membranes when GTP is bound (Fischer von Mollard et al., 1991; Sudhof, 2004). Removal of all four Rab3 proteins leads to perinatal lethality and abnormal short-term plasticity and vesicle dynamics in neuronal cultures (Schlüter et al., 2006). In Drosophila, Rab3 proteins are implicated in active zone dynamics. At the Drosophila neuromuscular junction, Rab3 deletion resulted in presynaptic terminals devoid of active zone proteins and calcium channels, which could be reversed within 24 h after re-introduction of Rab3 to mutant Drosophila larvae (Graf et al., 2009). Rab3 proteins interact with RIM1α in their GTP-bound form (Wang et al., 1997), and two Rab3 isoforms have been shown to be required for presynaptic long-term plasticity at various synapses. Rab3A deletion has been shown to abolish both presynaptic LTP and LTD at hippocampal mossy fiber-CA3 synapses (Castillo et al., 1997), as well as presynaptic LTP at cortical–lateral amygdala synapses (Tzounopoulos et al., 1998) and late phase LTP at CA3-CA1 synapses (Huang et al., 2005). Rab3B deletion has been shown to eliminate presynaptic LTD at inhibitory synapses in the hippocampus (Tsetsenis et al., 2011). Due to its interaction with RIM1α and their dual requirement for presynaptic LTP at excitatory synapses, Rab3 proteins are believed to function together with RIM1α during presynaptic long-term plasticity (Sudhof, 2004; García-Junco-Clemente et al., 2005; Kaeser et al., 2008a; Tsetsenis et al., 2011). However, Rab3A knockout mice and RIM1α knockout mice display different presynaptic LTD phenotypes at hippocampal mossy fiber-CA3 synapses (Tzounopoulos et al., 1998; Castillo et al., 2002), suggesting that Rab3A and RIM1α may play different roles in different forms of presynaptic long-term plasticity. How Rab3 proteins regulate presynaptic strength during plasticity and whether its interaction with RIM1α is required remain to be determined.

#### Munc13

Munc13 proteins (Munc13-1, Munc13-2, Munc13-3, Munc13-4) are active zone priming proteins required for neurotransmission. In the absence of full-length Munc13 proteins (Munc13-1 and Munc13-2 double KO mice), no vesicle priming was detected in hippocampal excitatory synapses of autaptic cultured pyramidal neurons, resulting in a complete loss of spontaneous and evoked synaptic transmission (Augustin et al., 1999; Rosenmund et al., 2002; Varoqueaux et al., 2002). Munc13 proteins have multiple functional domains. Current evidence supports an essential role for the MUN domain in vesicle priming (Basu et al., 2005; Stevens et al., 2005), a regulatory role for the C2A domain in vesicle priming (Betz et al., 2001; Deng et al., 2011), and roles for the C2 domains, C1 domain, and calmodulin (CaM)-binding sequence in short-term synaptic plasticity (Junge et al., 2004; Basu et al., 2007; Shin et al., 2010a). The C2A domain of Munc13 interacts with RIM1α. Disruption of this interaction resulted in autoinhibitory homo-dimerization of Munc13 which reduced the size of the readily releasable pool and evoked EPSC in cultured neurons (Betz et al., 2001; Deng et al., 2011). Munc13-1, but not Munc13-2, is required for presynaptic LTP at hippocampal mossy fiber synapses (Breustedt et al., 2010), suggesting different roles of the two full-length Munc13 proteins in long-term plasticity. Further, it has been shown that the requirement for Munc13-1 in hippocampal mfLTP is through its interaction with RIM1α, supporting regulation of vesicle priming as the cellular basis for long-term presynaptic plasticity (Yang and Calakos, 2011).

#### Voltage-gated calcium channels (VGCCs)

Calcium influx through VGCCs is an essential step in triggering fast synchronous neurotransmitter release. The magnitude and duration of calcium transients as well as the basal intracellular calcium concentration greatly shape synaptic release probability (Sudhof, 2004; Neher and Sakaba, 2008). Therefore, factors affecting intracellular calcium concentration are well suited to mediate a change in neurotransmitter release. Such factors include the open probability and open duration of VGCCs, the intracellular calcium stores, and the calcium buffers that shape the calcium microdomains. To date, only the calcium transients and the functions of VGCCs have been studied in the expression of presynaptic long-term plasticity. At nucleus accumbens synapses, where mGluR2/3- and eCB-LTD are present and may both function through suppression of the cAMP/PKA pathway, P/Q-type VGCCs are selectively inhibited to reduce neurotransmission in presynaptic LTD. At the plateau phase of agonist-induced mGluR2/3-LTD, the relative contributions of P/Q-type VGCC to field excitatory post-synaptic potentials were greatly reduced (Robbe et al., 2002a). Further, blockade of P/Q-type throughout the experiment, but not L- or N-type VGCCs, occluded agonist-induced mGluR2/3-LTD and tetanus-induced eCB-LTD (Robbe et al., 2002a; Mato et al., 2008). These findings indicate that presynaptic LTD in the nucleus accumbens is expressed by inhibition of P/Q-type VGCCs. Selective inhibition of P/Q-type VGCC has also been reported in presynaptic LTD at the mossy fiber-stratum lucidum interneuron synapses. At these synapses, calcium transients are reduced after HFS-induced LTD; this reduction is due to a reduction of calcium influx through the P/Q-type VGCCs, as shown by pharmacological experiments (Pelkey et al., 2006).

Presynaptic LTP, however, is not always associated with an enhancement of VGCCs. Multiple groups have shown with different calcium indicators that calcium transients are unaltered at the mossy fiber bouton after LTP expression (Regehr and Tank, 1991; Kamiya et al., 2002; Pelkey et al., 2006). At the mossy fiber-stratum lucidum interneuron synapses, where LTD results from a reduction in calcium influx, de-depression or LTP of the same synapse does not involve a reversal (i.e., increase) of the calcium transient, suggesting that LTP and LTD are not mere reciprocals of each other mechanistically (Pelkey et al., 2008). This observation also excludes the slight possibility that none of the calcium indicators were sensitive enough to detect a change in calcium transients before and after long-term plasticity, confirming that regulation of calcium transients does not appear to be involved in these forms of presynaptic LTP.

## Conclusions and future directions

Long-term forms of presynaptic plasticity are abundant and contribute unique computational features to circuit function. In this review, we have discussed methods typically used for identifying presynaptic mechanisms in long-term plasticity, those synapses at which this process has been observed, and our current understanding of the molecular mechanisms underlying presynaptic long-term plasticity. Historically, the necessity of using postsynaptic neurons as “reporters” of presynaptic activity has imposed an additional obstacle specific to studying presynaptic forms of long-term plasticity. Going forward, however, technical advances in the ability to image single synapses, vesicles and molecules are removing this obstacle. These technical advances hold great promise to accelerate our understanding of the detailed cellular and molecular mechanisms of presynaptic long-term plasticity. Major questions to resolve in the near future include identifying the specific synaptic processes and molecular events that produce an increase in release probability and discretely manipulating presynaptic long-term plasticity *in vivo* to demonstrate its behavioral significance in various circuits.

### Conflict of interest statement

The authors declare that the research was conducted in the absence of any commercial or financial relationships that could be construed as a potential conflict of interest.

## Footnotes

^1^Note that for CV to be independent of quantal size q, it is required that q remains constant for the repeated measurements needed to calculate CV. Rapid changes in q may come from postsynaptic receptor desensitization or trafficking. Moreover, it has been recently shown that CV is altered by crosslinking AMPA receptors to inhibit lateral diffusion (Heine et al., 2008). The possibility of such postsynaptic processes must be kept in mind in interpreting CV changes.
